# Profiling of Croatian Consumers Based on Their Intention to Consume Farmed Fish

**DOI:** 10.3390/foods11142158

**Published:** 2022-07-20

**Authors:** Greta Krešić, Elena Dujmić, Dina Lončarić, Snježana Zrnčić, Nikolina Liović, Jelka Pleadin

**Affiliations:** 1 Department of Food and Nutrition, Faculty of Tourism and Hospitality Management, University of Rijeka, Primorska 46, P.O. Box 97, 51410 Opatija, Croatia; gretak@fthm.hr (G.K.); nikolina.liovic@fthm.hr (N.L.); 2 Center for Projects, Faculty of Tourism and Hospitality Management, University of Rijeka, Primorska 46, P.O. Box 97, 51410 Opatija, Croatia; elena.dujmic@fthm.hr; 3 Department of Marketing, Faculty of Tourism and Hospitality Management, University of Rijeka, Primorska 46, P.O. Box 97, 51410 Opatija, Croatia; dinal@fthm.hr; 4 Laboratory for Fish Pathology, Croatian Veterinary Institute, Savska Cesta 143, 10000 Zagreb, Croatia; zrncic@veinst.hr; 5 Laboratory for Analytical Chemistry, Croatian Veterinary Institute, Savska Cesta 143, 10000 Zagreb, Croatia

**Keywords:** marine aquaculture, consumer segments, farmed fish, intention to consume, wild fish

## Abstract

Today’s increased demand and consumption of fish would be impossible to ensure without aquaculture. Farmed fish, however, is often considered inferior among consumers in comparison to its wild counterparts. The aim of this study was to profile Croatian fishery consumers based on their intention to consume farmed fish. The participants in this study were a nationally representative sample of people responsible for food purchasing within the household (n = 977), whose responses were collected by the CAWI (computer-aided web interviewing) method. Four clusters were identified and described: farmed fish enthusiasts (21.1%), farmed fish supporters (17.4%), indifferents (44.7%), and farmed fish sceptics (16.8%). Results showed that consumer segments differed significantly with respect to age, income, employment status, living region, and physical activity. Furthermore, intention to consume farmed fish is related to fish consumption in general (those with higher intention are more frequent fish consumers). Interestingly, prejudices against farmed fish are present in all clusters; however, these prejudices are more pronounced among those with the weakest intention to consume farmed fish. Differences between clusters were observed also in respect to product information and preferences, knowledge about fish, places of usual purchase, and source of information about fishery products. The obtained results could be used in designing marketing strategies to promote farmed fish consumption.

## 1. Introduction

Fish has been widely recognized as an integral part of a balanced and healthy diet, thanks to its high nutritional value and associated multiple health benefits. The recommended consumption of two servings a week ensures the provision of key nutrients: vitamin D, iodine, selenium, and especially polyunsaturated omega-3 fatty acids [1]. Moreover, fish plays an important role in correcting micronutrient deficient diets in developing countries, and thus not only can it reduce the prevalence of malnutrition, but it can also alter imbalanced high-calorie low-micronutrient diets in developed countries [2]. Regular fish consumption could also address today’s burden of non-communicable diseases, such as obesity, cardiovascular disease mortality, heart failure, stroke, depression, and mental illnesses [3,4,5].

Human population growth, rising incomes coupled with urbanization, and changes in dietary habits have contributed to the increased demand and consumption of fish [2,6,7,8]. Currently, world per capita fish consumption stands at 20.5 kg, which is more than double than in the 1960s, and has even surpassed population growth and the consumption of other animal proteins [2]. Given that more than 90% of the world’s marine fish stocks are fully exploited or overexploited, while at the same time the percentage of stocks within biologically unsustainable levels are continuously increasing, the current demand for fishery products would be impossible to ensure without aquaculture. Aquaculture accounts for more than half of fish production intended for human consumption, and it is expected to increase even further, considering it is the fastest-growing food-production industry in the world [2]. Due to its intensification, however, once traditional practices are now raising some environmental concerns owing to eutrophication and the use of problematic substances, such as hormones and antibiotics [9,10]. Therefore, the sustainable development of aquaculture is currently a priority among various global and national policymakers [2,11,12,13].

Although aquaculture is not necessarily perceived negative per se among consumers, they nevertheless have greater preferences for wild fish than farmed fish [14,15], especially in terms of quality, taste, health, nutritional value, and safety [16,17,18,19,20]. Such beliefs in the superiority of wild fish can negatively affect the consumption of farmed fish [21]. A recent study, however, has suggested that a positive perception of wild fish does not necessarily lead to its higher consumption. Despite the fact that wild fish was more preferred, farmed fish was consumed more often [17].

According to the recent data, Croatia is in first place (alongside Malta) among EU members with regard to overweight and obesity, with 65% of the adult population belonging to these categories [22], while 42% of total deaths are the results of cardiovascular diseases [23]. When asked about their self-perceived health status, only 60% of Croatians identified their health as very good or good [24]. One way to counteract these negative health trends is to revive traditional regional dietary patterns (e.g., the Mediterranean diet), which, when combined with a healthy lifestyle, can improve the health status of the population. One of the elements of such health-related dietary patterns is an increase in the consumption of fish and fishery products.

About 23% of the total production of fishery products in Croatia originates from aquaculture (21,771 t), while the rest comes from catches [25]. However, Croatia is in 7th place in terms of farmed finfish production in the EU, but also in 4th place in marine finfish farming. Among marine fish, sea bream and sea bass are the most produced species (7780 t, 6754 t, respectively), while carp is the most produced freshwater fish (1691 t). In total finfish production, marine aquaculture accounts for 87% [25]. Paradoxically, despite high production, Croatia is in 13th place among EU countries in terms of per capita fish consumption, and with an annual fish consumption of 20.8 kg per capita, it is below the EU average (24.0 kg) [26]. In the European Union, wild-caught products account for three-quarters of the total apparent consumption. In 2019, per capita consumption of wild-caught products amounted to 18.3 kg [26]. Therefore, more effort is needed to promote fish consumption, with special emphasis on farmed fish.

To the best of our knowledge, only a few studies have investigated fish consumption in Croatia [27,28,29,30] and only one study has focused on farmed fish, examining consumer knowledge and perception about fish from organic aquaculture [30]. In view of the above, the aim of this study is to identify segments of Croatian fishery consumers based on their intention to consume marine farmed fish and to detect similarities and differences among these segments according to their sociodemographic characteristics, fish consumption habits and preferences in at-home and away-from-home occasions, beliefs about wild and farmed fish, knowledge about fish, usual place of purchase, and sources from which they obtain information about products. The identification and profiling of consumer segments should help producers and marketers in planning marketing strategies that target various segments of consumers, with the aim to promote the consumption of marine farmed fish.

## 2. Materials and Methods

### 2.1. Subjects

The data collection fieldwork was performed in December 2019, by the professional market research agency Ipsos among its consumer panel members, using the CAWI (computer-aided web interviewing) method. Stratified random sampling and proportional quota in line with the national population distribution for gender, age, and region were performed, according to the latest estimates of the State Statistical Offices of Croatia. Participants were a nationally representative sample of people who are responsible for food purchasing within the household in Croatia. The initial sample size consisted of 1000 respondents, aged 18 to 65.

The final eligible study sample that could be considered as fishery product consumers was selected based on a positive response to the question: “Did you consume fishery products in the last 12 months?” The final study sample consisted of 977 participants.

### 2.2. Questionnaire Design

Data presented in this paper are part of the extended research conducted within the framework of the European project AdriAquaNet—*Enhancing Innovation and Sustainability in Adriatic Aquaculture* (AdriAquaNet, Interreg V-A Italy–Croatia 2014–2020 Program, Blue innovation, ID10045161, Grant Agreement No. 36008). The research instrument used for the purpose of this study is available in Supplementary Materials S1.

The name of the project was given to participants before the survey, and the meaning of the term “marine aquaculture” was clearly explained. The species of fish were specified in the questionnaire, which indicated that the focus of the research was on marine fish and that the terms “fishery products”, “fish” and “farmed fish” refer to marine fishery products and marine farmed fish. The questionnaire used for the purpose of this study included the following parts: sociodemographic characteristics and frequencies of physical activity, frequency of fish consumption at-home and away-from-home, intention to consume farmed fish, beliefs about wild and farmed fish, importance of product information, preferences for fishery products, objective and subjective knowledge, and place of purchase and source of information about fishery products. Filling out the questionnaire took about 20 min.

Sociodemographic characteristics included the participants’ gender, age, education level, number of household members, number of household members under the age of 18, employment status, region of living, and average household income per month. To assess the frequency of physical activity, participants were asked to report whether they engaged in a more intense workout (running, fast walking, aerobic, fitness, etc.) for at least 20 min 1 day or less in a week (inactive), 2–3 times (lightly active), 4–5 times (moderately active), or 6–7 times (vigorously active).

Fish consumption at-home and away-from-home was measured as the self-reported frequency of consumption of two generic categories of fish: white fish and fatty fish. This classification comes from their difference in nutritional composition, price, and sensory properties, which could be important for consumers, and it is common in several research papers [31,32,33]. Levels of consumption frequency at-home were encoded in seven rank ordinal categories: once a year or less, once in 3 months, 2–3 times a month, once a week, 2–3 times a week, 4–5 times a week, every day. Away-from-home fish consumption was measured with nine frequency categories: never, once a year or less, once in 6 months, once in 3 months, 2–3 times a month, once a week, 2–3 times a week, 4–5 times a week, almost every day. For the purpose of this study, never was calculated within the category of once a year or less. The sample size consisted of those who declared to eat in foodservice facilities at least once a year or less, which gave a total of 918 respondents.

Behavioural intention to consume farmed fish was measured with four formulated statements related to the future intention to either purchase or consume farmed fish at home or in catering facilities. The unidimensionality of the construct was checked by principal components analysis, while the reliability of the scales was determined by measuring the internal consistency coefficient, Cronbach’s alpha (α = 0.87).

Beliefs about wild and farmed fish were tested with 19 statements [16], in which respondents were asked to indicate the extent to which they agree with the proposed statements on a 5-point Likert scale, ranging from 1 (strongly disagree) to 5 (strongly agree).

Consumers’ knowledge about fish was measured through their objective, as well as subjective, knowledge. Objective knowledge was measured with 7 statements on a true/false scale, based on previous studies [34,35,36]. Two of the statements were false, “Fish is a source of dietary fibre” and “The sea bass and sea bream available in the European market are exclusively wild species”, while the remaining were true. The number of correct answers was summed for each participant, giving an aggregated score of objective knowledge on a scale of 0–7 [36]). Subjective knowledge was measured with 4 statements on a 5-point Likert scale (1 = strongly disagree, 5 = strongly agree), based on a previous study [16]. Internal reliability was verified calculating Cronbach’s alpha coefficient (α = 0.92), while unidimensionality of the construct was assessed by principal components analysis.

The importance of product information included 11 possible information cues: shelf life, production method, country of origin, previous freezing, processing method, quality label, list of ingredients, eco-label, nutritional value, and brand. A 5-point Likert scale was used, ranging from 1 (not at all important) to 5 (very important).

Furthermore, participants were asked about their preferred type of fishery products, where they had to indicate on a 5-point Likert scale the level of their preference (1 = strongly not prefer, 5 = strongly prefer) towards whole fresh fish, fresh cleaned fish, fresh fillets, frozen whole fish, frozen fillets, canned fish, smoked or dried fish, and fish products (sticks, burgers).

In order to determine consumers’ usual place of purchase of fishery products, multiple choice answers were given: shopping mall, local store, fish market, directly from fisherman, on the fish farm, self-fishing [37].

Sources for obtaining information about the importance of fishery products were measured by a multiple-choice question, with the following possible responses: television, radio, newspaper, internet, fisherman/salesman, friends/relatives/family, doctor, professional and scientific literature, personal experience [37].

The questionnaire was developed in English and further translated into Croatian by professional translators. The back-translation method was performed to ensure the quality and accuracy of the translation.

### 2.3. Data Analysis

Descriptive statistics were used to describe the sample and items included in the questionnaire. Cluster analysis was performed to identify the different segments of study participants based on their intention to consume farmed fish. Cluster analysis is a multivariate technique aimed at grouping objects based on their characteristics, and is used in a number of disciplines [38]. First, an agglomerative hierarchical cluster analysis using Ward’s method and squared Euclidean distance was utilized to determine the optimal number of clusters based on a dendrogram. Then, k-means clustering, a non-hierarchical method, was performed to form the final clusters. A combination of both methods is the approach recommended by many researchers to compensate for the weaknesses of one method with the advantages of the other [38]. The differences between the obtained segments were described using Welch’s ANOVA (one-way analysis of variance) with the Games–Howell post hoc test, since unequal variances between the clusters were established by Levene’s test (*p* < 0.05). Pearson’s chi-square test was applied to the categorical variables. Data were analysed using the statistical software IBM SPSS Statistics version 26 (IBM Corp., Armonk, NY, USA). The level of statistical significance was set at *p* < 0.05.

## 3. Results and Discussion

The cluster analysis technique has been commonly used to analyse the characteristics of food consumers since it allows segmenting into different homogenous groups to summarize consumer behaviour into a limited number of distinct consumer profiles [39]. A four-cluster solution according to the participants’ intention to consume farmed fish emerged as the optimum from cluster analysis. All statements were statistically different (*p* < 0.001) among the clusters (Table 1). Mean values of the average scores for intention to consume farmed fish were as follows: Cluster 1 (average score 4.25 (0.43), 21.1% participants), Cluster 2 (average score 3.34 (0.34), 17.4% participants), Cluster 3 (average score 2.95 (0.26), 44.7% participants), and Cluster 4 (average score 1.73 (0.47), 16.8% participants) (Table 1).

### 3.1. Sociodemographic Characteristics of Fish Consumers

Among the total sample of Croatian fishery consumers, there were no gender-specific differences and the average age of the participants was 42.61 years (data not shown). Almost half of the respondents (46.1%) had finished secondary school, while about half (52.5%) had earned a bachelor’s or master’s degree. Sixty-seven percent of the fish consumers were employed full time, and 13.8% were retirees. Although the average number of household members was three, more than half of the respondents (61%) had no children under the age of 18. According to average monthly income, almost 60% of the participants in Croatia were middle class, while 14% could be considered as being upper class. One third of the respondents (34.1%) were physically inactive, while 33.4% could be considered lightly physically active (2–3 days per week) (Table 2).

The obtained four clusters did not differ significantly according to gender, education level, number of household members, and number of children under the age of 18. Even though studies suggest that women are more likely to be farmed fish consumers [16,40,41] since they are in most cases responsible for procuring and preparing food for the household and, therefore, are more accustomed to products from aquaculture [16], this has not been confirmed in our study. The lack of gender-specific differences among clusters could be explained by the fact that in our study sample the persons responsible for food procurement were equally distributed by gender. Significant differences obtained in other sociodemographic characteristics will be described briefly.

Regarding age distribution, Cluster 1 had a higher percentage of consumers aged 31–40 in comparison with other clusters. Similarly, Cluster 3 consisted of relatively more consumers belonging to the youngest age group (18–30 years old; 24.3%) than other clusters. Cluster 2 consisted mainly of participants between 41–50 years of age (30.6%), while Cluster 4 had the highest share of consumers older than 51 (40.2%). In previous studies, age also emerged as one of the most important sociodemographic characteristics. Generally, younger consumers are more likely to consume farmed fish, whereas older consumers, with more traditional eating habits, have a greater preference for wild fish species [20,29,40,41,42]. This was partially confirmed by our study, since the highest intentions to consume farmed fish were evident in Clusters 1 and 2, which comprised of younger participants, while the exhibited intention was the lowest in Cluster 4, in which the participants were the oldest.

Although it would be expected that consumers with lower incomes would be more likely to consume farmed fish [42], given its lower price, it has been identified that high-potential aquaculture consumers have higher incomes [43], which corroborates with our findings. With regard to average household income, although the highest share of participants in all clusters belong to the middle class, Clusters 1 and 2 had a higher share of upper-class participants (about 18%). In addition to this fact, Cluster 2, in comparison with other clusters, also had a higher portion of participants belonging to the high class of income (10.6%). Cluster 4 had the highest share of lower-class participants (14%) based on monthly average household income.

Differences in employment status among the clusters were the most evident between Clusters 1 and 4. Significantly more consumers employed full-time belong to Cluster 1 (73.3%), whereas Cluster 4 had a significantly higher portion of retired (21.3%), unemployed (14.0%) persons, and students (9.8%), groups with expected lower income. Consequently, this cluster group had the lowest intention to consume farmed fish.

Place of residence can influence consumers’ preferences and consumption habits, since consumers living or raised in coastal regions prefer more wild fish and eat less farmed fish products [29,40]. In our study, the results do not provide a coherent conclusion regarding region of residence, mostly because more than one-quarter of all Croatian citizens live in the city of Zagreb. In our nationally representative sample, the majority of consumers in all clusters were from the capital city of Zagreb, similar to the total sample. Cluster 2, however, had a larger share of consumers from the Adriatic coastal region—South (28.8%)—a part of Croatia where the majority of fish farms are located, while Cluster 4 consisted of relatively more consumers from another Adriatic coastal region—South-West (19.7%). Cluster 3 had significantly more consumers living in the continental part of Croatia—North and East (34.7%)—than did other clusters.

Regarding frequencies of physical activity, it is evident that more consumers in Cluster 2 were physically inactive or lightly active (77.6%) compared with other clusters. Most consumers in the other three clusters, however, were also not very active, although Cluster 1 had a higher percentage of participants who were moderately active (26.2%), while Clusters 3 and 4 had a higher share of participants who were vigorously active (16.2% and 17.1%, respectively) (Table 2).

### 3.2. Frequencies of At-Home White and Fatty Fish Consumption

European consumers have a habit of consuming fish more often at home than away from home. Nearly two-thirds (64%) of European consumers consume fish at home at least once a month, while 33% consume it at least once a week [44].

**Figure 1:**
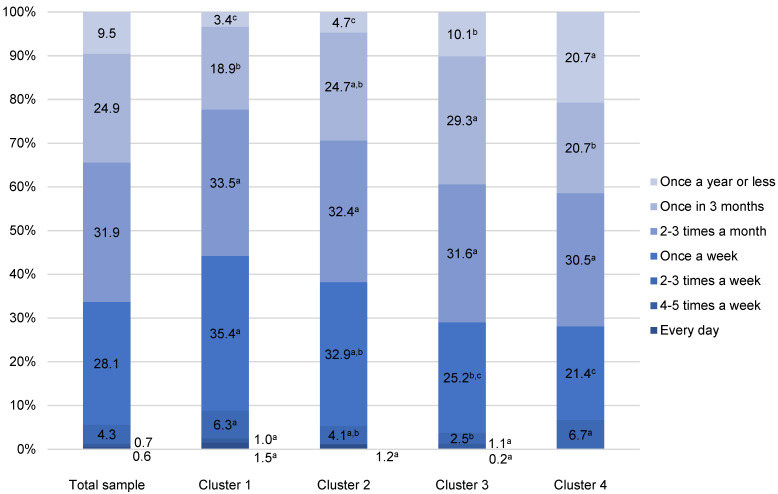
At-home white fish consumption of Croatian fishery consumers (n = 977). ^a–c^ Different letters in the same consumption frequency category indicate statistically significant differences (*p* < 0.001).

Among the total sample of Croatian fish consumers, the highest share consumed white fish at home 2–3 times a month (31.9%), followed by the frequency of once a week (28.0%) and once in 3 months (24.9%) (Figure 1). When at-home white fish consumption frequency is analysed according to clusters of consumers, and compared with the average frequencies of the total sample, it is obvious that the percentage of consumers who eat white fish once a week or more is the highest in Cluster 1 (44.2%), followed by Cluster 2 (38.2%). Clusters 3 and 4 consisted of less frequent consumers of white fish, since the percentage of those who consume it less than once a week is high (71.0% and 71.9%, respectively). However, Cluster 3 had a larger share of those consuming white fish once in 3 months (29.3%), while in Cluster 4 there were more participants who eat white fish once a year or less (20.7%) in comparison with other clusters (Figure 1).

**Figure 2:**
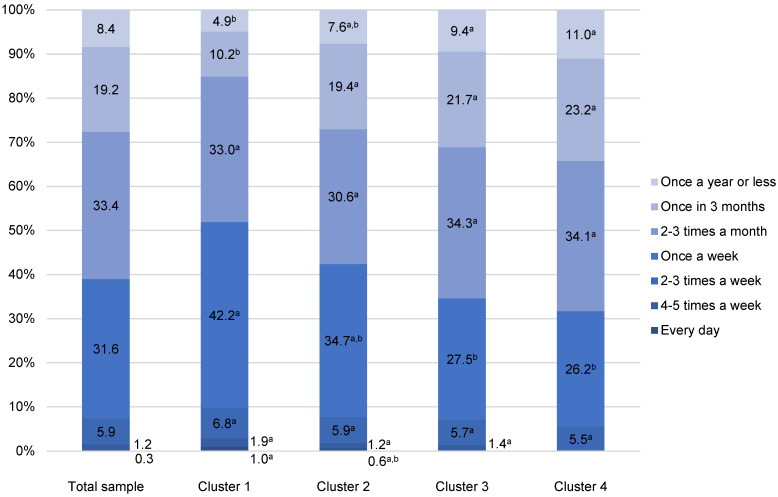
At-home fatty fish consumption of Croatian fishery consumers (n = 977). ^a,b^ Different letters in the same consumption frequency category indicate statistically significant differences (*p* = 0.005).

Frequency of at-home fatty fish consumption among Croatian fish consumers followed a similar pattern as in the case of white fish, as about 31.6% of participants consumed it once a week, while 60.9% consumed it less often (Figure 2). The heaviest consumers of fatty fish were again members of Cluster 1, with more than half of them (51.9%) eating it once a week or more often, whereas members of Cluster 2 were the second largest consumers of fatty fish (42.4%). Consumers in Clusters 3 and 4 consumed fatty fish less frequently (65.4% and 68.3%, respectively, consume it 2–3 times a month or less) (Figure 2).

Recent results of at-home fishery and aquaculture products consumption among consumers from 28 EU countries confirmed that one of the main reasons for buying or eating fish and aquaculture products is because they are healthy, while the biggest barrier to greater consumption is a lack of understanding of the information labelled on fishery and aquaculture products [45]. Health consciousness and dietary habits of consumers are closely related to frequency of physical activity [46,47]. Previously, it was found that consumers who engage in regular physical activity tend to consume seafood products more frequently [32]. However, our observation that more physically engaged fish consumers belonged mainly to Clusters 3 and 4, which represented participants with lower intention to consume farmed fish (Table 2), supported previous findings that respondents who are engaged in physical activity more than four times per week prefer to purchase wild fish [29].

From the results obtained in our study, it could be concluded that frequency of fish consumption decreases from Cluster 1 to Cluster 4, indicating that those who have higher intention to consume farmed fish are in fact more frequent fish consumers. This could be explained by the fact that Croatian fish consumers are price sensitive and that the price of fish influences their purchase habits, since they consider fish as an expensive food and farmed fish is cheaper compared with its wild counterparts (unpublished data). Contrary to us, the previously mentioned survey among consumers in 28 EU countries [45] confirmed that a higher frequency of fishery and aquaculture products consumption is closely related to wild product preferences. These same consumers, however, stated that they consider fish a cheap food, which also distinguishes them from our respondents. It is possible that the obtained differences are due to the large number of countries involved, which results in large differences among respondents.

### 3.3. Frequencies of Away-From-Home White and Fatty Fish Consumption

In today’s modern society, it is a common habit to eat out in foodservice facilities. Higher incomes, hectic lifestyles, greater urbanization, as well as more women in the workforce who do not have so much time for cooking, have majorly influenced this shift. At the same time, food service outlets are much more available and affordable than they were previously [48]. Households in the EU spent over EUR 630 billion in foodservice facilities in 2019, representing 7.1% of their total consumption expenditure [49]. Even though the spending of Croatian households in foodservice facilities is similar in percentage (6.1% of their total consumption expenditure), it represents only EUR 2.4 billion in total [49].

**Figure 3:**
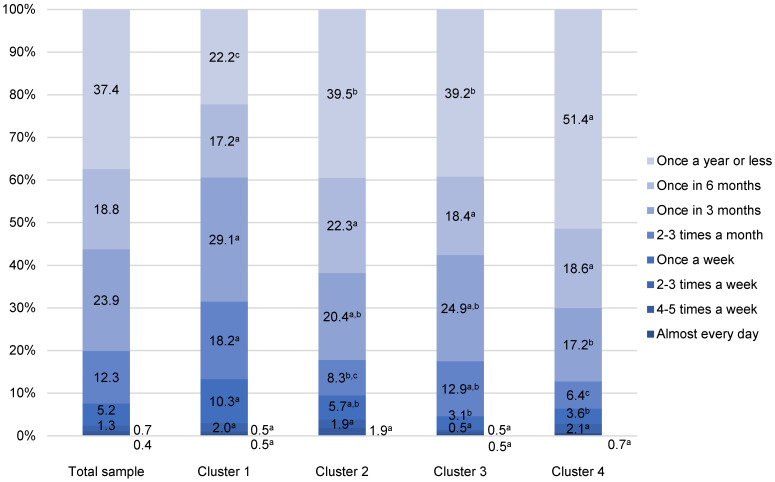
Away-from-home white fish consumption of Croatian fishery consumers (n = 918). ^a–c^ Different letters in the same consumption frequency category indicate statistically significant differences (*p* < 0.001).

**Figure 4:**
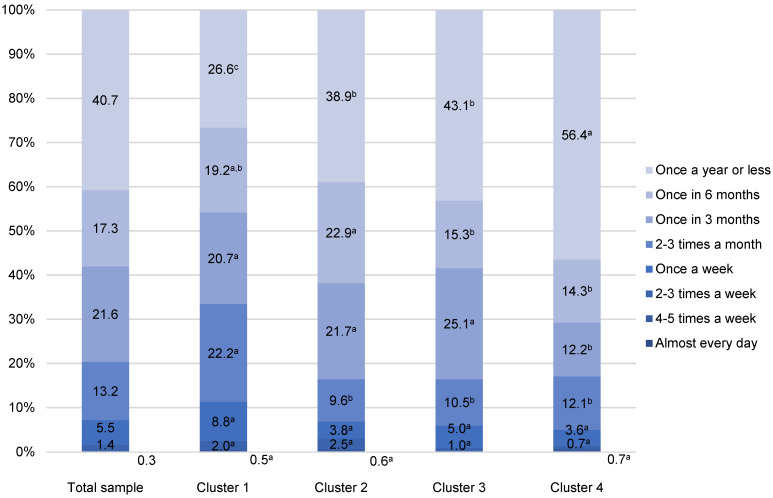
Away-from-home fatty fish consumption of Croatian fishery consumers (n = 918). ^a–c^ Different letters in the same consumption frequency category indicate statistically significant differences (*p* < 0.001).

Generally speaking, Croatian consumers eat fish in foodservice facilities quite rarely (Figure 3 and Figure 4). The highest share of participants eat white and fatty fish away-from-home once a year or less (37.4% and 40.7%, respectively). Among those who eat fish away-from-home more than once a year, the most often reported frequency is once in three months (23.9% for white fish and 21.6% for fatty fish) (Figure 3 and Figure 4).

The frequency of eating white fish away-from-home followed the segmentation according to the participants´ intention to consume farmed fish. The share of those who consume white fish once a year or less often increases from Cluster 1 (22.2%) to Cluster 4 (51.4%). In addition, Cluster 1 eats white fish away-from-home more often in comparison with the other three clusters, as 31.5% of the study participants consume it 2–3 times a month or more often. As expected, participants in Cluster 2 have an almost two times less frequent habit of away-from-home white fish consumption (2–3 times a month or more, 17.8%), since, as shown previously through the segmentation process, participants in this cluster express the lowest intention to consume farmed fish in foodservice facilities. Similarly, in Cluster 3, 17.5% of participants have a frequent habit of consuming white fish in foodservice facilities, whereas Cluster 4 has the lowest consumption (12.8%) (Figure 3).

As for white fish, the share of consumers who consume fatty fish away-from-home once a year or less increases from 26.6% (Cluster 1) to 56.4% (Cluster 4). Similarly, one-third of the members of Cluster 1 (33.0%) consume fatty fish in foodservice facilities 2–3 times a month or more often, whereas 16.5% of participants in Clusters 2 and 3 have the same habit. In Cluster 4, this share was similar to Clusters 2 and 3 (17.1%) (Figure 4).

The results of away-from-home fish consumption among clusters followed a similar pattern as at-home fish consumption, with the exception that Cluster 2 was more similar to Clusters 3 and 4, indicating their low consumption of fish in catering facilities.

In the European Union, a significant part of fishery products consumption is also consumed in various foodservice facilities, namely away-from-home. Twenty-one percent of Europeans consume fishery products away-from-home at least once a month, while 7% consume them away-from-home at least once a week [44]. As expected, our results showed that Croatian consumers eat more fish at home than away from home, similar to previous research [34]. The cited research confirmed that those consumers who have better recognized the quality of fish and are more knowledgeable about the characteristics and methods of preparation of fishery products consume it more frequently [34].

Our previous research [50], in which we split the total study sample according to their frequency of eating in foodservice facilities, confirmed that those who visit foodservice facilities more often also order white and fatty fish there more often. In addition, those who eat less often in foodservice facilities have more pronounced negative beliefs about farmed fish [50].

The characteristics of European consumers who more often eat fishery products away from home than at home are: born between 1961 and 1997, without children, with a university degree and high income [51]. This description is quite similar to the sociodemographic characteristics of our consumers in Cluster 1. However, regarding the method of fish production (wild vs. farmed), contrary to our results, a comprehensive study which includes consumers from 28 EU members confirmed that consumers who more often visit foodservice facilities preferred wild fish [52]. The reason for this deviation is probably again the fact that Croatian consumers choose farmed fish precisely because of the cheaper price in restaurants.

### 3.4. Beliefs about Wild and Farmed Fish

Croatian fish consumers generally believe that wild fish is superior in comparison with farmed fish, in the following characteristics: it has a healthier diet, is healthier, of better quality, more nutritious, tastes better, and is firmer. On the other hand, farmed fish is considered more artificial but easily available, with more guarantees and cheaper prices. Additionally, farmed fish is considered less safe, but at the same time, better controlled (Table 3).

From the segmentation of consumers, it can be seen that consumers in Cluster 4 hold strong positive beliefs about wild fish, while consumers in Clusters 1, 2 and 3 are more moderate in their positive opinions. Since all the belief statements were presented in the format of comparison wild vs. farmed fish, the strength of beliefs could be determined as a numerical value which expressed the level of agreement with statements.

Consumers in Cluster 1 had the most favourable belief that wild fish is more affected by parasites than farmed fish. These consumers more strongly disagreed with the statements that wild fish is fresher (2.93) and more available (2.24) compared to farmed fish, which could lead to the conclusion that they consider farmed fish to be fresher and more available. Surprisingly, they held equally strong beliefs about the superiority of wild fish in terms of quality and taste, as did Cluster 4. This observation could suggest that although this group of consumers have declared their intention to buy farmed fish, this intention is not based solely on their beliefs.

Consumers in Cluster 2 had a more favourable view of farmed fish with regard to the attributes of health, nutrition and control. Similar to consumers in Cluster 1, they believe farmed fish is fresher and more available. Interestingly, members of Cluster 3 tend be more restrained in their responses regarding all aspects of beliefs about wild and farmed fish, considering that their average scores are closer to midpoints in all statements in comparison with other clusters.

Consumers in Cluster 4 had the strongest beliefs regarding wild fish. This group of consumers did not believe that wild fish is more affected by parasites (2.67), and at the same time believed that wild fish has a healthier diet (3.84), is fresher (3.28), is more nutritious (3.70), and provides more guarantees (3.62). The highest values, obtained as the level of agreement of consumers in this group with the offered statements, confirm the strength of their beliefs (Table 3). Furthermore, they most strongly believed that farmed fish was more artificial.

Generally, all four clusters have similar opinions regarding the safety of farmed fish, considering it less safe in comparison with wild fish, more affected by marine pollution and parasites, and containing more heavy metals and antibiotics. In previous studies, farmed fish scored higher regarding these parameters [16,17]. It has been scientifically proven that the risk of containing harmful substances is lower in farmed fish, since wild fish may ingest them from the environment they live in [53,54]. However, consumers’ perception of wild fish being more resistant to chemical and microbial contamination may stem from the idea of the better well-being of wild fish [19]. Contrary to that, farmed fish were seen as offering more control through the production process, while at the same time being more handled. Indeed, fish farms have the ability to control the production process and more easily manage the presence of toxic contaminants [55].

Wild fish scored high in different quality dimensions among all four clusters, especially with regard to overall quality and taste. In previous studies, consumers also agreed that wild fish were of better quality and had a better taste [16,20,56,57,58]. Uncertainty about fish feed may influence consumers’ perception of the lower quality and taste of farmed fish [56], given the obtained results that wild fish is perceived as having a healthier diet. Interestingly, in blind experiments, when the information about the production method was not provided, consumers preferred farmed fish more, whereas, conversely, a greater preference for wild fish was found when production method information was provided [18,59,60].

Croatian consumers perceived wild fish as being healthier and more nutritious, which is in line with the results obtained in previous studies [16,17,20,61]. These differences between the two types of fish, however, are minimal in Clusters 2 and 3, compared with Cluster 4. Such beliefs are not based on actual scientific facts [62], since farmed fish has more constant nutritional composition as it is less affected by seasonal variations than wild fish is, and their dietary intake can also be adjusted [55]. One of the common concerns among consumers is that farmed fish is fattier than wild fish. This concern was the most evident among consumers in Clusters 2 and 4. Indeed, farmed fish have higher total lipid content because of their regular feeding and lower physical activity; however, due to the highest share of total fat, the amounts of beneficial omega-3 fatty acids may even be higher in comparison with those in the same quantity of wild fish [55,63,64], which suggests that farmed fish can be as beneficial as wild fish, particularly in terms of potential to prevent cardiovascular diseases [55]. Consumers’ perceptions of wild fish being healthier may originate from the idea that healthy foods are more natural [65], i.e., less artificial. This strong belief in the artificiality of farmed fish is evident among all four clusters, even though Cluster 4 holds the strongest belief. In addition, consumers may idealize traditional fishing as a natural way of obtaining fish [66].

All consumer segments agreed that farmed fish is cheaper, with no significant differences among the clusters. Although the high price of fish is generally one of the barriers to fish consumption [15], in the case of farmed fish, consumer perception of it being cheaper compared with wild fish is one of the motivators for its consumption [61].

Contrary to other European consumers [16,17,42], Croatian consumers do not seem to make any distinctions between wild and farmed fish with regard to freshness. Upon consumer segmentation, the most important conclusion drawn from the analysis pertains to the fact that Clusters 1 and 2, which have the intention of buying and consuming farmed fish in the future, consider farmed fish to be fresher. Indeed, they have accurate beliefs since farmed fish is usually fresher at the time of purchase because of the greater control over its production process [67].

### 3.5. Importance of Product Information

Croatian consumers are quite interested in product information, as confirmed by the high values for importance (in the range of 3.29 to 4.45, on a scale of 1–5). The top five pieces of information according to the level of importance they have for Croatian consumers are as follows: shelf life (4.45), country of origin (4.21), if the product was previously frozen (4.18), quality label (3.97), and information about production method (wild or farmed fish) (3.82). Consumers in Cluster 1 are much more interested in product information compared with consumers in the other clusters (Table 4). In this group of consumers, the information which is significantly more important to them than to consumers in other clusters includes: country of origin (4.48), information if the product was previously frozen (4.46), information about production method (wild or farmed) (4.02), and eco-labels (3.88). Cluster 4 and Cluster 1 equally value information about shelf life, product brand, and quality label, while Cluster 3 can be said to contain the least interested consumers (Table 4).

A number of studies have shown consumers’ preference and willingness to pay a premium price for eco-labelled fish [14,68]. The highest interest in eco-labels was demonstrated in Cluster 1, which suggests that this segment of consumers not only have the strongest desire to consume farmed fish, but they are also highly sensitive to ecological and environmental features when buying fish. A recent study conducted in Croatia, however, revealed that only half of the study participants are willing to pay a higher price for fresh fish with an organic label, and half of them are even unaware of the possibility to buy such fish [30]. This points out the need for better communication with and education of consumers as to what a sustainable eco-label is [69]. Although an eco-label is important to consumers, it is not of the highest importance in purchase decisions [70,71]. Similar to our study, several review studies have confirmed that the country of origin is the most important attribute in consumers’ choice of fish [14,15,68] and, accordingly, marketers of aquaculture products are advised to prominently display the country of origin. There is a clear preference for domestic products vs. imported ones due to several factors: shorter transportation distance, considering fish is a highly perishable product; health and safety concerns about products from foreign countries; more trust in local products; as well as a high sense of ethnocentrism in consumers [14,15,68]. Therefore, to build a sustainable image, aquaculture should highlight the locality of the products as an important aspect of sustainability. Indeed, the country-of-origin label (“produced in own country”), together with an eco-label, functions the best as a driver of consumers’ fish choice [72].

### 3.6. Preferences for Fishery Products

Croatian consumers mostly preferred fresh fishery products. Their ranking of preferences was as follows: cleaned fish (3.97), whole fish (3.80), and fillets (3.70). Frozen products are less preferred, while the lowest preferences were for smoked or dried (2.27), canned (2.28), and fish products (i.e., burgers, sticks, etc.) (2.49) (Table 5). It is not surprising that fresh fish is much more preferred than other preserving methods since other studies have also confirmed European preference towards chilled fish [14,15,40,73], and handling and processing evokes in consumers the perception of loss of quality, safety, and nutritional value [15].

Significant differences were found between Clusters 1 and 4 in preferences for fish products. While consumers in Cluster 1 are more open to all types of fish products (including frozen, canned, and processed), consumers in Cluster 4 are much more conservative and reported lower preferences for all products. They especially do not prefer smoked and dried products (1.88). Clusters 2 and 3 did not differ significantly with regard to preferences for fishery products (Table 5).

### 3.7. Knowledge about Fish

As previous studies have confirmed the important role of knowledge in fish consumption behaviour [34,35,74,75], we wanted to explore whether there are any differences among different clusters of consumers. A distinction should be made, however, between subjective knowledge (how much a person thinks they know) and objective knowledge (how much a person actually knows) [76].

The results of the score obtained for objective knowledge confirm that Croatian consumers are quite knowledgeable (5.6 out of 7). They do not feel as confident in their knowledge, however, as the average score for subjective knowledge was around midpoint (3.05 out of 5) (Table 6).

Participants in Cluster 1 have a significantly higher level of both objective (5.74) and subjective (3.46) knowledge in comparison with Cluster 4, whose members possess the lowest level of both types of knowledge (5.42 and 2.88, respectively). Clusters 2 and 3 exhibited similar objective and subjective knowledge, as did the total sample (Table 6).

Previous researchers have found that a higher level of subjective knowledge is present among consumers who eat fish more frequently [34,35,75], and that subjective knowledge is a stronger predictor of fish consumption behaviour [74,75,77]. This corroborates our findings, since the highest fish consumption frequency was among Cluster 1 consumers, who also exhibited the highest levels of subjective knowledge. Yet, in our study, objective knowledge also proved to be a considerable factor influencing fish consumption, since members of Cluster 4 are characterized as the least knowledgeable and the lightest fish consumers. However, this conclusion should be taken with caution since their level of objective knowledge can still be considered as high.

### 3.8. Place of Purchase

In a recent study conducted in four Mediterranean European countries (France, Spain, Greece, and Italy), fishmongers and specialised stores were the most preferred places to buy fish, while supermarkets were places of the second rank of importance [73]. Croatian consumers often buy fishery products at fish markets (78.1%) and in shopping malls (68.4%), and it seems that they share similar preferences regarding the place of purchase as consumers from other Mediterranean countries.

Consumers in Cluster 1 have the habit of buying fishery products in shopping malls (75.7%), but also at fish farms (17.0%), while for consumers in Cluster 4, it is evident that they more often buy fishery products directly from fishermen (32.3%), as well as having the habit of fishing (25.6%). The fact that Cluster 4 contains a significant proportion of retirees, who also have lower incomes and live in the coastal part of Croatia, coincides with their habit of fishing more often compared with the other clusters. It stands to reason that people who fish, or who buy fish from fishermen, prefer wild fish, thanks to their experience. Although one study confirmed that consumers who engage in recreational fishing activities consume seafood products more often [78], this was not confirmed in our study. Place of purchase for consumers in Cluster 2 and Cluster 3 did not differ from the total study sample (Table 7).

### 3.9. Source of Information

It is worth exploring the type of information consumers are interested in with regard to fishery products, the sources they use, and whether this kind of information meets their expectations and intention to use [36]. Croatian consumers use different sources for obtaining information about fishery products (Table 8). The most often used were the internet (70.9%) and television (61.8%). Half of the total study sample used friends, relatives, and family members (47.8%), as well as personal experience (54.9%) as sources of information.

The clusters differed significantly in the frequency of internet usage, newspapers, and personal experience. Consumers in Cluster 1, relative to consumers in Cluster 4, used the internet (81.6 vs. 60.4%), newspapers (34.5 vs. 24.4%), and personal experience (64.1 vs. 53.7%) significantly more often. It could be concluded that Cluster 1 uses the most diverse sources of information, as a result of their increased interest in topics related to nutrition, which could consequently lead to better nutrition knowledge as previously discussed. Clusters 2 and 3 follow the same pattern for information usage, as does the total study sample (Table 8).

Similar results were obtained by the previously mentioned study among Mediterranean fish consumers, which showed that unofficial sources of information are much more preferred than institutional ones, and in which high values were obtained for personal experience and habits [73]. The participants in our study, however, displayed a greater preference for the internet and television, whereas personal experience was the most preferred source of information in those countries [73].

### 3.10. Cluster Profiling

Based on the participants´ intention to consume farmed fish, the resulting clusters were labelled as follows: farmed fish enthusiasts (Cluster 1), farmed fish supporters (Cluster 2), indifferents (Cluster 3), and farmed fish sceptics (Cluster 4).

Cluster 1—Farmed fish enthusiasts are mainly 31–40 years old, moderately physically active, with a higher share of those from the upper class of income. They are the heaviest consumers of white and fatty fish on all occasions. Although these consumers believe that farmed fish is fresher and more available than wild fish, they hold strong positive beliefs of the superiority of wild fish in terms of quality and taste. This group is the most interested in product information, particularly in the country of origin, information if the product was previously frozen, production method (wild or farmed), and eco-label. They are more open to purchasing all types of fishery products. This group, which is the most knowledgeable compared with the other clusters, usually buys fishery products in shopping malls, but also on fish farms. They use various sources of information, but mostly internet, newspapers, and personal experience.

Cluster 2—Farmed fish supporters are 41–50 years old, physically inactive, living in the coastal part of Croatia (south). The highest share of high-income participants is in this cluster. They have weaker habits of fish consumption compared with Cluster 1. These consumers consider farmed fish to be healthier, more nutritious, and more controlled. This cluster is quite similar to Cluster 3 regarding the importance of product information, preferences for fishery products, knowledge about fish, place of purchase, and sources of information.

Cluster 3—Indifferents is the biggest group of consumers and contains more young people (18–30) than the average. Consumers from this group are of middle class income, and are mainly from the continental part of Croatia. Frequency of at-home and away-from-home fish consumption is lower compared with Clusters 1 and 2. Members of this cluster tend to be more restrained in their responses regarding all aspects of beliefs about wild and farmed fish. Interest in product information is the lowest in this group.

Cluster 4—Farmed fish sceptics is the smallest cluster, but has the oldest participants (51–60 years old), a high proportion of unemployed persons, retirees, and students, with the lowest income, living in the coastal part of Croatia (south-west). Their frequency of at-home and away-from-home eating fish is the lowest among all clusters. This group holds the strongest positive beliefs regarding wild fish. Similar to Cluster 1, they value information about shelf life and quality. They are much more conservative and reported lower preferences for all types of fishery products (they especially do not prefer smoked and dried products). This group uses limited sources of information and has the lowest level of knowledge about fish. They often buy fishery products directly from fishermen, but also have a habit of fishing.

## 4. Conclusions and Policy Implication

This research provides valuable information that policy makers and marketers can use to encourage farmed fish consumption among Croatian consumers. Most consumers eat fish at home, while in away-from-home occasions it is rarely eaten. It can be seen that, although there are prejudices against farmed fish among all consumer clusters, these prejudices are still deeply rooted among older residents. They are less interested in consuming fish and have less knowledge about fish. Generally, consumers who have more knowledge about fish also consume more fish. This points to the importance of informing and educating consumers about the nutritional value of fish and health benefits of fish consumption. Consumers´ beliefs about farmed fish could change if consumers’ information and knowledge about farmed fish is improved. Therefore, policymakers and marketing managers are encouraged to develop a strategy to inform and educate the population about the health benefits of fish in general, and farmed fish in particular. Such a campaign should aim to increase knowledge about farmed fish. Since the Internet and television are the primary sources of information for consumers, these communication channels should be used.

Because consumers think farmed fish is cheaper than wild fish, policymakers and marketing managers can use this in a promotional campaign to help consumers in decision-making. For example, they can explain to consumers that they can provide themselves and their families with a nutritionally valuable and healthy meal for less money. Furthermore, food policymakers and marketing managers should provide consumers with important information when buying fish, such as information about shelf life, country of origin, if the product was previously frozen, quality label, and information about production method (wild or farmed fish).

Although the largest number of consumers buy fish at fish markets, a relatively large proportion buy fish in shopping malls. Therefore, it is crucial to make farmed fish available to consumers in convenient places for them to buy. For example, as it is not convenient for employed consumers to buy fish at fish markets, a broader range of products should be made available to them in shopping malls. Furthermore, even though Croatian consumers prefer fresh fishery products, it has been found that those who consume the most fish also eat other fish-based products. Therefore, consumers should be offered a variety of products based on farmed fish that will be easy to prepare.

Finally, it is important to note that eating habits are acquired from an early age when it comes to fish prejudices and habits. Therefore, policymakers are encouraged to create strategies aimed at schools and kindergartens to give young people the opportunity to acquire farmed fish-eating habits that they will maintain throughout their lives.

According to the legal framework, aquaculture in the Republic of Croatia is a strategic economic sector, and therefore is part of other development strategies. The aquaculture sector, however, is still far from reaching its full growth potential and meeting the growing demand for more sustainable marine foods. The primary objectives of aquaculture development in the future should focus on increasing productivity and the resilience of aquaculture production to climate change, strengthening the competitiveness of the aquaculture sector, restoring rural and coastal economies and improving livelihoods in rural and coastal areas, and promoting innovation in the aquaculture sector [79].

This is the first study on the intention to consume farmed fish conducted in Croatia on a nationally representative sample of people responsible for buying food for the household. An additional strength of this study is that it takes into account overall fish consumption (at-home and away-from-home). Precise consumer segmentation can be used to implement targeted measures aimed at individual consumer groups, with the aim of increasing fish consumption. Since the share of farmed fish on the Croatian market is small, when examining fish consumption, we did not distinguish between farmed and wild fish. This can be considered a limitation of this study, and, in the future, the focus should be on the consumption of farmed fish instead of overall fish consumption.

## Figures and Tables

**Table 1:** Intention to consume farmed fish among Croatian fishery consumers (n = 977).

Intention to Consume Farmed Fish	Cluster 1 (n = 206)	Cluster 2 (n = 170)	Cluster 3 (n = 437)	Cluster 4 (n = 164)	*p*
In the future I plan to buy farmed fish	4.29 (0.62)	3.81 (0.57)	2.92 (0.46)	1.82 (0.75)	<0.001
In the future I plan to consume farmed fish in catering facilities	4.21 (0.56)	2.66 (0.64)	2.95 (0.60)	1.49 (0.62)	<0.001
I will certainly buy farmed fish	4.25 (0.64)	3.84 (0.57)	2.87 (0.44)	1.88 (0.82)	<0.001
I will certainly consume farmed fish in catering facility	4.26 (0.62)	3.06 (0.83)	3.06 (0.56)	1.71	<0.001
Average score (SD)	4.25 (0.43)	3.34 (0.34)	2.95 (0.26)	1.73 (0.47)	<0.001

Values are expressed as means (SD).

**Table 2:** Sociodemographic characteristics of study sample (n = 977).

Sociodemographic Variables		Total Sample (n = 977)	Cluster 1 (n = 206)	Cluster 2 (n = 170)	Cluster 3 (n = 437)	Cluster 4 (n = 164)	*p*
**Gender (%)**	Female	49.6	47.1	44.1	51.5	53.7	0.234
	Male	50.4	52.9	55.9	48.5	46.3	
**Age (%)**	18–30	21.5	17.0	20.0	24.3	21.3	
	31–40	22.6	27.7	20.6	23.3	16.5	0.035
	41–50	25.0	24.3	30.6	24.3	22.0	
	51–65	30.9	31.1	28.8	28.1	40.2	
**Education level (%)**	Primary school or lower	1.4	1.0	1.2	1.6	1.8	
	Secondary school	46.1	47.1	45.9	44.9	48.2	0.972
	Bachelor’s, master’s or higher	52.5	51.9	52.9	53.5	50.0	
**Number of household members;** Mean (SD)		3.29 (1.33)	3.48 (1.47)	3.26 (1.32)	3.25 (1.30)	3.20 (1.24)	0.142
**Number of household members under the age of 18;** Mean (SD)		0.62 (0.91)	0.74 (0.95)	0.59 (0.89)	0.60 (0.91)	0.56 (0.89)	0.182
**Employment status (%)**	Employed full time	67.3	73.3	70.0	69.1	52.4	0.012
	Part-time employee	2.8	2.4	2.9	3.0	2.4	
	Unemployed	8.7	7.3	8.8	7.3	14.0	
	Retiree	13.8	12.1	10.6	13.0	21.3	
	Student	7.4	4.9	7.6	7.6	9.8	
**Region of living (%)**	City of Zagreb	30.0	29.1	30.0	30.2	30.5	
	North	14.2	14.6	13.5	16.9	7.3	
	East	14.5	12.6	10.6	17.8	12.2	0.001
	Centre	7.3	7.8	8.8	6.6	6.7	
	South-west	13.5	13.1	8.2	13.5	19.5	
	South	20.5	22.8	28.8	14.9	23.8	
Average household income per month (%) *	Lower	8.3	6.8	7.1	7.3	14.0	0.008
	Middle	59.6	61.2	52.9	62.0	57.9	
	Upper	14.1	18.0	18.8	11.7	11.0	
	High	6.3	5.3	10.6	5.3	6.1	
	N/A	11.7	8.7	10.6	13.7	11.0	
**Physical activity (%)**	1 day or less	34.1	30.6	38.2	35.7	29.9	<0.001
	2–3 days	33.4	35.9	39.4	28.8	36.0	
	4–5 days	20.0	26.2	17.1	19.2	17.1	
	6–7 days	12.6	7.3	5.3	16.2	17.1	

* Lower: HRK < 5000 (EUR < 667.7); Middle: HRK 5001–15,000 (EUR 667.8–2003.2); Upper: HRK 15,001–20,000 (EUR 2003.3–2670.9); High: HRK > 20,001 (EUR 2671.0); N/A, not applicable; HRK, Croatian currency (EUR 1 = HRK 7.5).

**Table 3:** Beliefs about wild and farmed fish of Croatian fishery consumers (n = 977).

Statement	Total Sample	Cluster 1	Cluster 2	Cluster 3	Cluster 4	*p*
Safety						
Wild fish is safer than farmed fish	3.12 (1.04)	3.06 (1.19)	3.09 (1.00)	3.08 (0.88)	3.35 (1.26)	0.084
Wild fish is more affected by marine pollution than farmed fish	2.96 (1.08)	3.07 (1.31)	2.90 (1.10)	3.00 (0.88)	2.80 (1.20)	0.150
Wild fish contains more heavy metals than farmed fish	2.83 (1.00)	2.84 (1.26)	2.84 (0.97)	2.85 (0.81)	2.77 (1.09)	0.873
Wild fish contains more antibiotics than farmed fish	2.22 (1.09)	2.12 (1.23)	2.22 (1.02)	2.33 (0.99)	2.07 (1.19)	0.057
Wild fish is more affected by parasites (*Anisakis*) than farmed fish	2.90 (0.94)	3.08 ^a^ (1.16)	2.85 ^a,b^ (0.89)	2.92 ^a^ (0.78)	2.67 ^b^ (1.03)	0.003
Wild fish has a healthier diet than farmed fish	3.65 (1.05)	3.76 ^a,b^ (1.12)	3.62 ^a,b^ (1.03)	3.54 ^a^ (0.94)	3.84 ^b^ (1.23)	0.008
Wild fish is healthier than farmed fish	3.59 (1.08)	3.63 ^a,b^ (1.17)	3.54 ^a^ (1.04)	3.48 ^a^ (0.97)	3.87 ^b^ (1.21)	0.003
**Quality**						
Wild fish is of better quality than farmed fish	3.77 (1.06)	3.87 ^a^ (1.07)	3.78 ^a,b^ (1.03)	3.61 ^b^ (0.98)	4.04 ^a^ (1.21)	<0.001
Wild fish is fresher than farmed fish	3.05 (1.13)	2.93 ^a^ (1.24)	2.88 ^a^ (1.09)	3.09 ^a,b^ (1.00)	3.28 ^b^ (1.29)	0.008
Wild fish is more nutritious than farmed fish	3.41 (1.08)	3.44 ^a,b^ (1.19)	3.29 ^a^ (1.15)	3.34 ^a^ (0.92)	3.70 ^b^ (1.22)	0.004
Wild fish is more fatty than farmed fish	2.29 (1.17)	2.27 ^a,b^ (1.37)	2.16 ^a^ (1.10)	2.44 ^b^ (1.04)	2.07 ^a^ (1.27)	0.001
Wild fish tastes better than farmed fish	3.78 (1.06)	3.91 ^a^ (1.11)	3.78 ^a,b^ (1.00)	3.62 ^b^ (0.96)	4.05 ^a^ (1.21)	<0.001
Wild fish is firmer than farmed fish	3.57 (1.05)	3.77 ^a^ (1.11)	3.51 ^a,b^ (1.02)	3.46 ^b^ (0.92)	3.70 ^a,b^ (1.25)	0.002
**Control**						
Wild fish is more controlled than farmed fish	2.53 (1.05)	2.44 ^a,b^ (1.24)	2.35 ^a^ (1.02)	2.62 ^b^ (0.92)	2.62 ^a,b^ (1.12)	0.012
Wild fish is more handled than farmed fish	2.88 (1.02)	2.96 (1.21)	2.85 (1.04)	2.88 (0.88)	2.79 (1.12)	0.562
Wild fish is more artificial than farmed fish	1.99 (1.07)	1.96 ^a,b^ (1.27)	1.99 ^a^ (1.02)	2.11 ^a^ (0.98)	1.69 ^b^ (1.05)	<0.001
Wild fish provides more guarantees than farmed fish	3.28 (1.09)	3.24 ^a^ (1.25)	3.08 ^a^ (1.03)	3.25 ^a^ (0.93)	3.62 ^b^ (1.26)	<0.001
**Purchase**						
Wild fish is easier to find than farmed fish	2.39 (1.10)	2.24 ^a^ (1.31)	2.26 ^a^ (1.12)	2.52 ^b^ (0.94)	2.36 ^a,b^ (1.15)	0.006
Wild fish is cheaper than farmed fish	2.47 (1.19)	2.38 (1.35)	2.34 (1.24)	2.56 (1.04)	2.47 (1.28)	0.128

Values are expressed as means (SD); ^a,b^ Different letters in the same row indicate statistically significant differences (*p* < 0.05).

**Table 4:** Importance of product information for Croatian fishery consumers (n = 977).

Information	Total Sample	Cluster 1	Cluster 2	Cluster 3	Cluster 4	*p*
Shelf life	4.45 (0.84)	4.60 ^a^ (0.79)	4.46 ^a,b^ (0.87)	4.32 ^b^ (0.86)	4.60 ^a^ (0.77)	<0.001
Nutritional value	3.51 (1.07)	3.53 (1.14)	3.42 (1.08)	3.54 (0.95)	3.51 (1.26)	0.639
List of ingredients	3.72 (1.05)	3.92 ^a^ (1.10)	3.69 ^a,b^ (1.07)	3.61 ^b^ (0.96)	3.79 ^a,b^ (1.16)	0.004
Country of origin	4.21 (0.94)	4.48 ^a^ (0.71)	4.21 ^b^ (0.95)	4.05 ^b^ (0.92)	4.26 ^a,b^ (1.14)	<0.001
Information about production method (wild or farmed fish)	3.82 (1.00)	4.02 ^a^ (0.98)	3.75 ^b^ (0.97)	3.71 ^b^ (0.95)	3.96 ^a,b^ (1.16)	<0.001
Product brand (producer)	3.61 (1.05)	3.79 ^a^ (1.03)	3.55 ^a,b^ (1.04)	3.49 ^b^ (1.00)	3.76 ^a^ (1.15)	0.002
Processing method	3.81 (0.96)	4.12 ^a^ (0.88)	3.78 ^b^ (0.94)	3.65 ^b^ (0.90)	3.86 ^a,b^ (1.11)	<0.001
Quality label	3.97 (0.93)	4.17 ^a^ (0.90)	3.96 ^a,b^ (0.90)	3.84 ^b^ (0.90)	4.10 ^a^ (1.04)	<0.001
Eco-label	3.63 (1.07)	3.88 ^a^ (1.07)	3.64 ^a,b^ (1.00)	3.53 ^b^ (0.99)	3.55 ^b^ (1.27)	<0.001
If the product was previously frozen	4.18 (0.94)	4.46 ^a^ (0.82)	4.21 ^b^ (0.97)	4.03 ^b^ (0.90)	4.22 ^a,b^ (1.06)	<0.001
Recommended method of preparation	3.29 (1.09)	3.33 (1.16)	3.19 (1.09)	3.29 (0.99)	3.32 (1.21)	0.645

Values are expressed as means (SD); ^a,b^ Different letters in the same row indicate statistically significant differences (*p* < 0.05).

**Table 5:** Preference for type of products among Croatian fishery consumers (n = 977).

Type of Product	Total Sample	Cluster 1	Cluster 2	Cluster 3	Cluster 4	*p*
Fresh, whole	3.80 (1.32)	4.15 ^a^ (1.17)	3.74 ^b^ (1.32)	3.68 ^b^ (1.32)	3.76 ^b^ (1.42)	<0.001
Fresh, cleaned	3.97 (1.15)	4.16 ^a^ (1.11)	4.02 ^a,b^ (1.06)	3.94 ^a,b^ (1.10)	3.76 ^b^ (1.34)	0.017
Fresh fillets	3.70 (1.21)	3.77 (1.30)	3.65 (1.10)	3.76 (1.13)	3.47 (1.35)	0.074
Frozen, whole	2.89 (1.23)	3.11 ^a^ (1.26)	2.88 ^a^ (1.25)	2.95 ^a^ (1.19)	2.43 ^b^ (1.20)	<0.001
Frozen fillets	3.36 (1.18)	3.55 ^a^ (1.13)	3.34 ^a^ (1.16)	3.45 ^a^ (1.11)	2.90 ^b^ (1.34)	<0.001
Canned	2.88 (1.19)	3.16 ^a^ (1.17)	2.81 ^b^ (1.14)	2.85 ^b^ (1.18)	2.66 ^b^ (1.24)	<0.001
Smoked or dried fish	2.27 (1.19)	2.51 ^a^ (1.29)	2.44 ^a,b^ (1.17)	2.24 ^b^ (1.15)	1.88 ^c^ (1.10)	<0.001
Fish products (sticks, burger)	2.49 (1.26)	2.62 ^a^ (1.33)	2.44 ^a,b^ (1.22)	2.56 ^a^ (1.20)	2.21 ^b^ (1.34)	0.014

Values are expressed as means (SD); ^a,b^ Different letters in the same row indicate statistically significant differences (*p* < 0.05).

**Table 6:** Knowledge about fish of Croatian fishery consumers (n = 977).

Knowledge	Total Sample	Cluster 1	Cluster 2	Cluster 3	Cluster 4	*p*
Objective knowledge	5.60 (0.97)	5.74 ^a^ (0.88)	5.61 ^a,b^ (0.88)	5.59 ^a,b^ (1.02)	5.42 ^b^ (1.04)	0.021
Subjective knowledge	3.05 (0.97)	3.46 ^a^ (0.94)	3.04 ^b^ (0.87)	2.94 ^b^ (0.92)	2.88 ^b^ (1.11)	<0.001

Values are expressed as means (SD); ^a,b^ Different letters in the same row indicate statistically significant differences (*p* < 0.05).

**Table 7:** Place of usual purchase of fishery products of Croatian fishery consumers (n = 977).

Place *	Total Sample (%)	Cluster 1 (%)	Cluster 2 (%)	Cluster 3 (%)	Cluster 4 (%)	*p*
In shopping mall	68.4	75.7 ^a^	75.9 ^a^	68.0 ^b^	52.4 ^c^	<0.001
In local store	14.6	18.9 ^a^	14.7 ^a,b^	14.9 ^a^	8.5 ^b^	0.047
At the fish market	78.1	84.5	78.8	75.1	77.4	0.062
Directly from fisherman	23.8	21.8 ^b,c^	26.5 ^a,b^	19.2 ^c^	32.3 ^a^	0.005
On the farm	9.2	17.0 ^a^	14.1 ^a^	5.5 ^b^	4.3 ^b^	<0.001
Self-catch	14.8	16.0 ^b^	8.8 ^c^	12.6 ^b,c^	25.6 ^a^	<0.001

* multiple answers; ^a–c^ Different letters in the same row indicate statistically significant differences (*p* < 0.05).

**Table 8:** Source of information about fishery products for Croatian fishery consumers (n = 977).

Source *	Total Sample (%)	Cluster 1 (%)	Cluster 2 (%)	Cluster 3 (%)	Cluster 4 (%)	*p*
Television	61.8	65.0	65.9	59.3	60.4	0.327
Internet	70.9	81.6 ^a^	75.3 ^a,b^	68.2 ^b,c^	60.4 ^c^	<0.001
Radio	16.2	18.9	16.5	14.2	17.7	0.438
Newspaper	28.1	34.5 ^a^	34.1 ^a,b^	24.3 ^c^	24.4 ^b,c^	0.009
Fisherman/salesman	28.2	28.2	25.3	29.1	29.3	0.811
Friends/relatives/family	47.8	52.9	47.6	46.7	44.5	0.378
Doctor	26.5	28.6	23.5	26.5	26.8	0.738
Professional and scientific literature	27.0	29.1	27.6	27.0	23.8	0.713
Personal experience	54.9	64.1 ^a^	53.5 ^b^	51.5 ^b^	53.7 ^b^	0.026

* multiple answers; ^a–c^ Different letters in the same row indicate statistically significant differences (*p* < 0.05).

## Data Availability

The datasets generated and analysed during the current study are not publicly available, but may be available from the corresponding author upon reasonable request.

## Supplementary Materials

The following supporting information can be downloaded at: https://www.mdpi.com/article/10.3390/foods11142158/s1, S1: Questionnaire.
