# Supplementary material for: Profiling of Croatian Consumers Based on Their Intention to Consume Farmed Fish
Source: Foods. 2022 Jul 20;11(14):2158. doi: 10.3390/foods11142158 (PMC9319490; doi:10.3390/foods11142158)
Supplement: Supplementary file 1 [file foods-11-02158-s001.zip › foods-1800340-supplementary.pdf]

Questionnaire S1

1. *How often do you personally consume following fishery products at home?*

Type of fishery product	Every day	4-5 times a week	2-3 times a week	Once a week	2-3 times a month	Once in 3 months	Once a year or less
White fish (sea bream, sea bass, hake and other)							
Fatty fish (sardine, mackerel, chub mackerel, tuna and other)							

2. *When eating in catering facility, how often the meal includes:*

	Almost every day	4-5 times a week	2-3 times a week	Once a week	2-3 times a month	Once in 3 months	Once in 6 months	Once a year or less	Never
White fish (sea bream, sea bass, hake and other)									
Fatty fish (sardine, mackerel, chub mackerel, tuna and other)									

3. *We kindly ask you to indicate the level of your agreement with the following statements.*

No.	Statement	Strongly DISAGREE				Strongly AGREE
1.	In the future I plan to buy farmed fish.	1	2	3	4	5
2.	In the future I plan to consume farmed fish in catering facilities.	1	2	3	4	5
3.	I will certainly buy farmed fish.	1	2	3	4	5
4.	I will certainly consume farmed fish in catering facility.	1	2	3	4	5

4. *We kindly ask you to indicate the level of your agreement with the following statements.*

No.	Statements	Strongly DISAGREE				Strongly AGREE
1.	Wild fish is safer than farmed fish.	1	2	3	4	5
2.	Wild fish is more affected by marine pollution than farmed fish.	1	2	3	4	5
3.	Wild fish contains more heavy metals than farmed fish.	1	2	3	4	5
4.	Wild fish contains more antibiotics than farmed fish.	1	2	3	4	5
5.	Wild fish is more affected by parasites (anisakis) than farmed fish.	1	2	3	4	5
6.	Wild fish has a healthier diet than farmed fish.	1	2	3	4	5
7.	Wild fish is healthier than farmed fish.	1	2	3	4	5
8.	Wild fish is of better quality than farmed fish.	1	2	3	4	5
9.	Wild fish is fresher than farmed fish.	1	2	3	4	5
10.	Wild fish is more nutritious than farmed fish.	1	2	3	4	5
11.	Wild fish is more fatty than farmed fish.	1	2	3	4	5
12.	Wild fish tastes better than farmed fish.	1	2	3	4	5
13.	Wild fish is firmer than farmed fish.	1	2	3	4	5
14.	Wild fish is more controlled than farmed fish.	1	2	3	4	5
15.	Wild fish is more handled than farmed fish.	1	2	3	4	5
16.	Wild fish is more artificial than farmed fish.	1	2	3	4	5
17.	Wild fish provides more guarantees than farmed fish.	1	2	3	4	5
18.	Wild fish is easier to find than farmed fish.	1	2	3	4	5
19.	Wild fish is cheaper than farmed fish.	1	2	3	4	5

5. When buying fishery products, how important is the availability of the following information to you? We kindly ask you to indicate the level of importance on the scale.

No.	The information	Not at all IMPORTANT				Very IMPORTANT
1.	Shelf life	1	2	3	4	5
2.	Nutritional value (the content of energy, proteins, fats and other nutrients)	1	2	3	4	5
3.	List of ingredients	1	2	3	4	5
4.	Country of origin	1	2	3	4	5
5.	Production method (wild vs farmed)	1	2	3	4	5
6.	Product brand	1	2	3	4	5
7.	Processing method (canning, special packaging, smoking etc.)	1	2	3	4	5
8.	Quality label	1	2	3	4	5
9.	Eco-label	1	2	3	4	5
10.	Previous freezing	1	2	3	4	5
11.	Recommended method of preparation	1	2	3	4	5

6. When buying fishery products, what type of products do you prefer?

Type of product	Strongly NOT PREFER				Strongly PREFER
Fresh, whole	1	2	3	4	5
Fresh, cleaned	1	2	3	4	5
Fresh, fillets	1	2	3	4	5
Frozen, whole	1	2	3	4	5
Frozen, fillets	1	2	3	4	5
Canned	1	2	3	4	5
Smoked or dried fish	1	2	3	4	5

Type of product	Strongly NOT PREFER				Strongly PREFER
Fish products (sticks, burgers)	1	2	3	4	5

7. *We kindly ask you to indicate if the answer is true or false.*

No.	Statement	TRUE	FALSE
1.	Fish is a source of dietary fibre.		
2.	Fish is a source of omega-3 fatty acids.		
3.	It is recommended to eat fish at least twice a week.		
4.	Consumption of fatty fish is important in the prevention of some chronic diseases, such as cardiovascular diseases.		
5.	High maternal fish consumption during pregnancy and infant's fish intake in the first year improves child developmental skills.		
6.	The sea bass and sea bream available in the European market are exclusively wild species.		
7.	The eyes of the fish demonstrate its freshness.		

8. *We kindly ask you to indicate the level of your agreement with the following statements.*

No.	Statement	Strongly DISAGREE				Strongly AGREE
1.	I consider that I know more about fish than the average person.	1	2	3	4	5
2.	I think that I know more about fish than my friends.	1	2	3	4	5
3.	I have a lot of knowledge about how to prepare fish.	1	2	3	4	5
4.	I have a lot of knowledge about how to evaluate the quality of fish.	1	2	3	4	5

9. *Where do you usually buy fishery products? (multiple choice)*

   a) In shopping mall
   b) In local store
   c) At the fish market
   d) Directly from fisherman
   e) On the farm
   f) Self-fishing

10. *From which sources did you get information about importance of fishery products consumption? (multiple choice)*

   a) Television
   b) Radio
   c) Newspaper
   d) Internet
   e) Fisherman/salesman
   f) Friends, relatives, family
   g) Doctor
   h) Professional and scientific literature
   i) Personal experience and acquired habits

Socio-demographic profile of respondents

1.	Age:	
2.	Sex:	a) Male<br>b) Female
3.	Number of household members:	
4.	Number of household members under the age of 18:	
5.	Average household income per month	a) Less than 5 000 HRK / Less than 667.7 EUR<br>b) 5 001 - 10 000 HRK / 667.8 - 1335.5 EUR<br>c) 10 001 - 15 000 HRK / 1335.6 - 2003.2 EUR<br>d) 15 001 - 20 000 HRK / 2003.3 - 2670.9 EUR<br>e) More than 20 001 HRK / 2671.0 EUR<br>f) Prefer not to say
6.	Education level:	a) Primary school or lower<br>b) Secondary school<br>c) Bachelor, master or higher

7.	Employment status:	a) Employed full time<br>b) Part-time employee<br>c) Student<br>d) Retiree<br>e) Unemployed
8.	How many days per week are you doing intense physical activity for at least 20 min (running, fast walking, aerobic, fitness etc.):	a) 1 day or less<br>b) 2 - 3<br>c) 4 - 5<br>d) 6 - 7
9.	Region of living:	a) Zagreb (City of Zagreb)<br>b) North Croatia (North)<br>c) Slavonia (East)<br>d) Lika and Banovina (Centre)<br>e) Istra, Rijeka and Primorje (South-west)<br>f) Dalmatia (South)
